# Notes and Notices

**Published:** 1887-08

**Authors:** 


### NOTES AND NOTICES.

A GOLDEN RULE.—"Study, learn, prescribe. If the low dilutions fail you, *don't be afraid to go up*."—*Eggert.*

FREQUENT REPETITION NOT ADVANTAGEOUS.—The careful homœopathic physician will not venture to repeat a dose of the same remedy, at short intervals, because no advantage is derived from this practice, but more frequently, as is attested by accurate observation, it is a source of certain injury.—*Hahnemann.*

STILL WAITING.—Two human skeletons, in sitting posture, have been found imbedded in solid rock in Arizona. It is supposed that they were a couple of prehistoric Internationalists who sat down to relate their wonderful cures to each other, and became so much interested that they didn't notice that it was growing late.—*Med. Era.* Not so; they were two Institute men sitting out in the cold waiting for "recognition."

THE FLY: THE GREAT DISINFECTANT!—The particular office of flies appears to be the consumption of those dead minute animals whose decaying myriads would otherwise poison the air. It was a remark of Linnæus that three flies would consume a dead horse sooner than a lion could. He, of course, included the families of the three flies. A single fly, the *Naturalist* tells us, will sometimes produce twenty thousand larvæ, each of which, in a few days, may be the parent of another twenty thousand, and thus the descendants of these flies would soon devour an animal much larger than a horse.—*Scientific American.*

BRIEF BUT ATTRACTIVE.—In the announcement of one of the "truly homœopathic" colleges our land is blessed with, we read: "During the opening week of the session attractive evening lectures on the Institutes of Homœopathy will be delivered by distinguished members of the profession."—One week given to Homœopathy, twenty-one to eclecticism!!

OVER-DOSING.—The smallest homœopathic dose, when properly selected and applied, effects wonders. It not unfrequently occurs that patients are overwhelmed, by incompetent homœopathists, with rapid succession of remedies, which, though well selected, and of the high potence, yet produce a state of such excessive irritability that the life of the patient is placed in jeopardy, and another dose, however mild, may prove fatal.—*Hahnemann.*

THE *Southern Journal of Homœopathy*, under the editorship of brother Fisher, continues to be one of our most interesting exchanges, We hope it may meet with the greatest success in its endeavor of introducing Homœopathy into the Southern States. In a recent issue we read two interesting papers by Drs. J. G. Gundlach and J. R. Mackenzie.

